# Exosomes in osteoarthritis and cartilage injury: advanced development and potential therapeutic strategies

**DOI:** 10.7150/ijbs.41637

**Published:** 2020-03-31

**Authors:** Quanfa Zhou, Youzhi Cai, Yangzi Jiang, Xiangjin Lin

**Affiliations:** 1 Department of Orthopaedic and Center for Sports Medicine, the First Affiliated Hospital, School of Medicine, Zhejiang University, Zhejiang, Hangzhou, China; 2 Dr. Li Dak Sum & Yip Yio Chin Center for Stem Cell and Regenerative Medicine, School of Medicine, Zhejiang University, Hangzhou, China.; 3 Institute for Tissue Engineering and Regenerative Medicine, School of Biomedical Sciences, Faculty of Medicine, The Chinese University of Hong Kong, Hong Kong S.A.R., China

**Keywords:** exosome, osteoarthritis, cartilage, exosomal RNA, stem cells, cellular and exosomal therapies

## Abstract

Articular cartilage injury is a common clinical problem, which can lead to joint dysfunction, significant pain, and secondary osteoarthritis (OA) in which major surgical procedures are mandatory for treatment. Exosomes, as endosome-derived membrane-bound vesicles, participating in intercellular communications in both physiological and pathophysiological conditions, have been attached great importance in many fields. Recently, the significance of exosomes in the development of OA has been gradually concerned, while the therapeutic value of exosomes in cartilage repair and OA treatment has also been gradually revealed. The functional difference of different types and derivations of exosomes are determined by their specific contents. Herein, we provide comprehensive understanding on exosome and OA, including how exosomes participating in OA, the therapeutic value of exosomes for cartilage injury/OA, and related bioengineering strategies for future therapeutic design.

## Introduction

The occurrence of osteoarthritis (OA) implies an imbalance in degradation-synthesis of chondrocytes, extracellular matrix (ECM) and subchondral bone, but the detailed molecular mechanisms remain unclear. Exosomes, as a type of extracellular vesicles (EVs), play a role in tissue-tissue and cell-cell communications in homeostasis and diseases, and the mechanism of exosomal involvement in the development of OA has only recently been reported. Thus, understanding how exosomes participate in the process of OA will help to seek a novel way for developing the treatment of OA.

Traditional non-surgical treatments for OA can improve symptoms but cannot restore articular cartilage regeneration or modify degenerative processes 1. Although surgical arthroplasty results in long-term functional improvement and improves quality of life, this only suit for the end stage of the diseases, and instability and infection are the most common limitations, necessitating further revision surgery. Cell-based therapy, especially mesenchymal stem cells (MSCs), has facilitated rapid advances in regenerative medicine for OA/cartilage injury in recent years. However, the clinical applications of stem cells have raised considerable concerns, such as teratomas, immune rejection, non-stable between batches and dose-dependent effect 2-3. The bio-effects of MSCs mainly through paracrine, especially *via* the exosomes they produced 4. Thus, exosome-based therapy may be a promising substitute for stem cell therapy on cartilage injury/OA, which provides the possibility of “cell-free” therapy.

This review will focus on the biological characteristics of exosomes, and the involvement of exosomes in pathology of OA will be discussed in detail. We will also discuss the evidence that shows how exosomes can be used as “cell-free” therapy for OA/cartilage injury, and the detailed exosome-based tissue engineering strategies. Finally, we will also discuss future development in this exciting field.

## The biological characteristics of exosomes

Exosomes are endosome-derived membrane-bound vesicles with diameters of 50-150 nm, released by cells in all living systems in physiological and pathophysiological conditions 5. Exosomes originate from the endosomes that are generated by endocytosis of the cytoplasmic membrane. Later, inside the cell, cargoes such as mRNA or proteins accumulate inside the endosomes, thus forming multivesicular endosomes or intraluminal vesicles 6. After further processing, exosomes are finally released through membrane fusion 7. At early ages, exosomes were regarded as useless cellular metabolic waste, but it has since been recognized that exosomes carry proteins, lipids, and nucleic acids, including mRNA, microRNA (miRNA), and long non-coding RNA, which play important roles in intercellular communications and cellular immune response 8.

The biogenesis and release of exosomes is a complex symphony involving a series of factors, representatively including the endosomal sorting complexes required for transport (ESCRT), ALIX (also known as programed cell death 6-interacting protein, PDCD6IP), phospholipase, vacuolar protein sorting-associated 4 (VPS4), Rab GTPase proteins, sphingomyelinase and ceramide. Recently, the inhibition effect of exosome release was reported by sustained activation of mechanistic target of rapamycin complex 1 (mTORC1) in both cells and animal models, while inhibition of mTORC1 stimulated the release of exosomes, which occurred concomitantly with autophagy 9.

The recognition of membrane receptors is the basis for the exosome-cell reaction and interactions. The activation of the receptors and subsequently lead to the activation of associated signaling pathways, subsequently the fusion of exosomes with plasma membrane or the endocytosis of exosomes occurs. Through above-mentioned internalization pathways, exosomes can either release their cargos into the recipient cells to exert their functions, or be directly degraded by lysosome for recycling 10. Thus, the function and biological characteristic of exosomes are determined by their specific contents.

## Pathological related exosomes in OA

**Figure 1:**
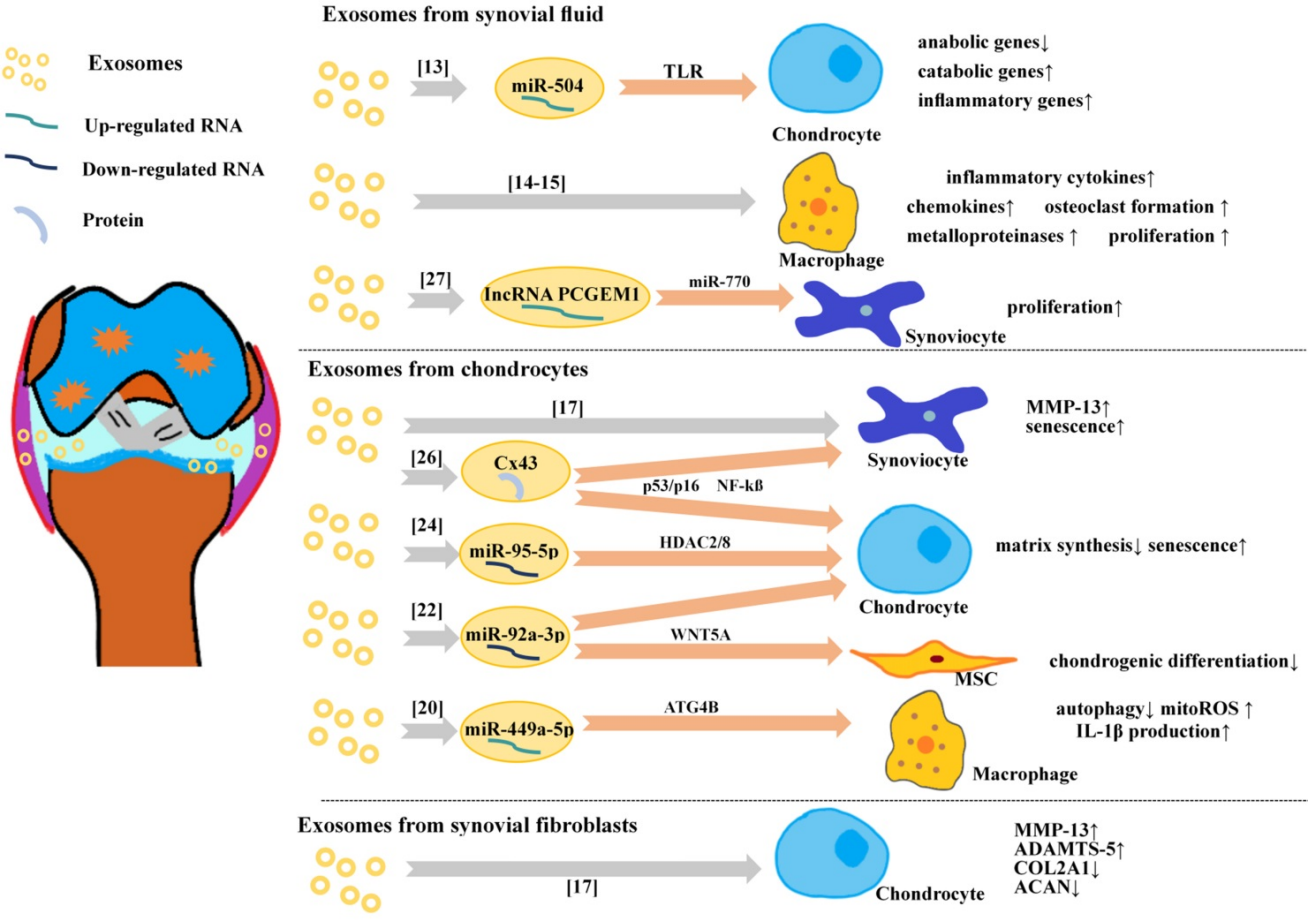
Exosomes involved in pathology of OA.

Although many beneficial biological effects are based on exosomes, in OA joint, chondrocytes, synoviocytes and immune cells may also deliver pathogenic signals to each other through exosomes, and these communications break the balance of joint microenvironment and further aggravate OA. In OA tissues, an increased number of immune cells associated with pro-inflammatory cytokine expression, including tumor necrosis factor (TNF)-α, interleukin (IL)-1β, IL-6 and IL-22, was detected from OA synovial tissue 11, while matrix metalloproteinase (MMP), is thought to be the major mediator of ECM breakdown, which causes the majority of the pathologies seen in OA 12. Analysis the relationship between exosomes and these important pathological factors may provide insights for our understanding of OA pathology (**Figure 1**).

### Exosomes in OA pathology

Exosomes in OA joint fluid were firstly analyzed by many research groups and it indeed usually links with pathological effects. Exosomes from OA joint fluid could influence the gene expression of chondrocytes negatively. Kolhe *et al* treated healthy articular chondrocytes with OA-derived exosomes, showing decreased anabolic genes expression and elevated expression of catabolic and inflammatory genes 13. In addition, exosomes from OA joint fluid could activate inflammatory cells. Domenis *et al* found that synovial fluid-derived exosomes significantly stimulated the release of several inflammatory cytokines, chemokines and metalloproteinases by M1 macrophages 14. Incubating macrophages with exosomes from synovial fluid of OA, Song *et al* demonstrated that the exosomes could induced proliferation and osteoclast formation without macrophage colony-stimulating factor (M-CSF) and receptor activator of nuclear factor kappa-B ligand (RANKL) 15.

Furthermore, some researches have studied the role of exosomes in the communication between chondrocytes and other cells in OA. Nakasa *et al* reported that when exosomes derived from chondrocytes treated with IL-1β were applied to fibroblast-like synoviocytes, there was a nearly three-fold increase in MMP-13 production as compared with exosomes derived from chondrocytes without IL-1β stimulation 16. In turn, Kato *et al* found exosomes from IL-1β stimulated human synovial fibroblasts (SFB) up-regulated MMP-13 and ADAMTS-5 expression in articular chondrocytes and down-regulated collagen alpha 1 (Col2a1) and aggregated proteoglycan core protein (ACAN) *in vivo* 17. In one chip-assay study, 50 miRNAs were identified in exosomes in response to IL-1β-stimulated SFB compared to in non-stimulated SFB, and among them, miR-4454 and miR-199b are related to inflammatory stimulation 18 and cartilage formation 19, respectively. Ni *et al* found the exosome-like vesicles from IL-1β-pretreated chondrocytes could promote mature IL-1β production of macrophages 20.

Kolhe *et al* reported the differential expression of miRNAs between exosomes from OA synovial fluid and normal synovial fluid 13. They found the gender differences of the expression of exosomal miRNAs, with only one (miRNA-504) existing in both genders. Interestingly, the authors also demonstrated that female OA-specific exosomal-miRNAs from synovial fluid were estrogen-responsive and targeted toll-like receptor (TLR) signaling pathways, which might relate to the increased prevalence of OA in post-menopausal females. Furthermore, the patients with OA had lower levels of exosomal miR-193b in plasma than normal control subjects 21.

The differential expression of miRNA of exosomes from chondrocytes in the inflammatory microenvironment which related to OA has been reported recently. Exosomal miR-92a-3p expression was significantly reduced in the OA chondrocyte-secreted exosomes. Mao *et al* found that miR-92a-3p suppressed the activity of a reporter construct containing the 3'-UTR and directly targeted WNT5A in both MSCs and chondrocytes 22, and WNT5A plays important role in both chondrogenic differentiation and cartilage degradation 23. Mao *et al* also found another exosomal miRNA (miR-95-5p) was down-regulated in OA chondrocytes 24. Furthermore, they demonstrated that miR-95-5p could regulate cartilage development and homoeostasis by directly targeting histone deacetylase (HDAC)2/8. HDAC2/8 tends to impede cartilage development by inhibiting the expression of cartilage‐specific genes 25.

Many exosomal miRNAs are founded related to OA development, there are exosomal proteins related to OA too. Recently, Varela-Eirín *et al* found that overexpression of channel protein connexin43 (Cx43) in chondrocytes increased senescence and exosomal Cx43 levels 26. In this study, OA chondrocytes showed increased levels of Cx43 within their EVs in comparison to the EVs isolated from healthy donors.

### The Diagnostic Value of Exosomes for OA

Theoretically, the variation levels of exosomal miRNAs or proteins mentioned above have potential to become biomarkers for OA diagnose. However, the value of exosomes as a diagnostic tool of OA is still under discussion. Recently, Zhao *et al* investigated the diagnostic value of exosomes from plasma and from synovial fluid in patients with OA in distinguishing the early stage of OA from progressive stage of OA 27. They found that in synovial fluid, the expression of exosomal lncRNA PCGEM1 was markedly higher in late-stage OA than in early-stage OA, and markedly higher in early-stage OA than normal controls, demonstrating that exosomal lncRNA PCGEM1 from synovial fluid might be a powerful indicator in distinguishing early-stage from late-stage OA. IncRNA PCGEM1 acts as a sponge lncRNA targeting miR-770 and then stimulates proliferation of osteoarthritic synoviocytes 28. However, the general evaluation of plasma and synovial fluid exosome was not viable for identifying OA stages, and the clinical application of exosomes in the diagnosis of OA remains challenging.

## Exosomes with therapeutic effects for OA and cartilage injury

### The exosomes from mesenchymal stem cells

**Figure 2:**
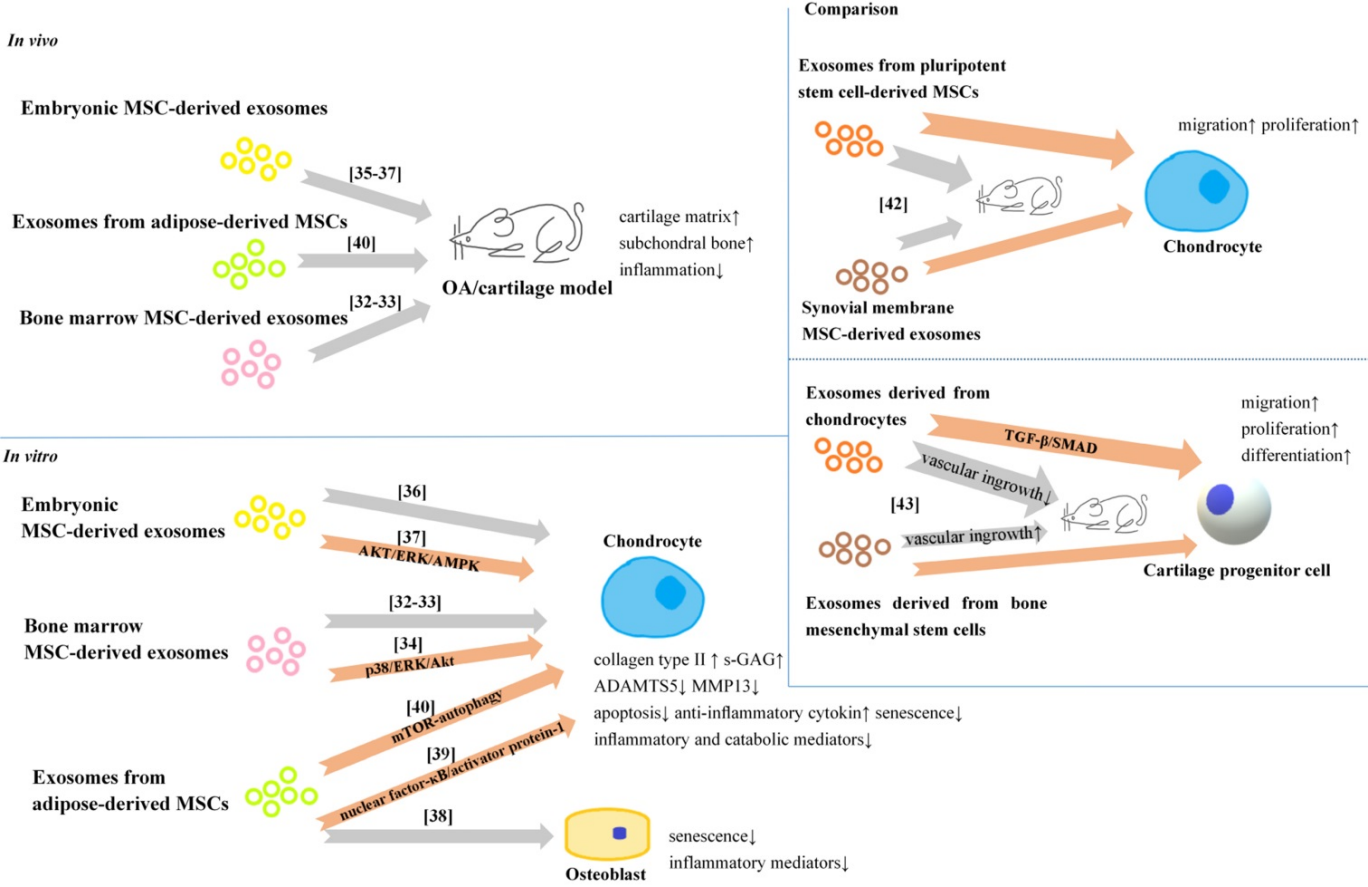
Exosomes from unmodified cells for OA therapy/cartilage.

Exosomes are one of the key secretory products of MSCs, resembling the effect of parental MSCs, and can be directly used as therapeutic agents for various disease models, such as cutaneous wound^29^, osteonecrosis of the femoral head 30, and neurological injury^31^. Recent years, exosomes from different unmodified MSCs were reported to have exact therapeutic effects on OA and cartilage injury (**Figure 2**).

#### Exosomes from different types of MSCs

Cartilage regeneration from bone marrow mesenchymal stem cells (BMSCs) is the core of microfracture technology. Exosomes from BMSCs have been studied in recent years. Cosenza *et al* demonstrated the protective effect of exosomes from BMSCs in the collagenase-induced OA model 32. Furthermore, they found exosomes from BMSCs could restore the expression of chondrocyte markers (type II collagen, aggrecan) while inhibiting catabolic (MMP-13, ADAMTS5) and inflammatory (iNOS) markers in OA-like chondrocytes *in vitro*. Zhu *et al* also revealed that BMSCs derived exosomes could protect chondrocytes from apoptosis and senescence 33. Furthermore, Qi *et al* observed the uptake of exosomes from BMSCs by chondrocytes 34, and exosomes from BMSCs could inhibit mitochondrial- induced apoptosis of chondrocytes in response to IL-1β, with p38, ERK, and Akt pathways involved.

Exosomes from embryonic MSC (ESCs) have shown the potential of alleviating matrix degradation and promoting cartilage repair in some animal models 35-37. Further *in vitro*, exosomes from ESCs could maintain the chondrocyte phenotype by increasing collagen type II synthesis and decreasing ADAMTS5 expression 36. Zhang *et al* demonstrated that the joint repair effects of exosomes from ESCs could be attributed to adenosine activation of protein kinase B(AKT), extracellular signal-regulated kinase (ERK) and adenosine monophosphate-activated protein kinase (AMPK) signaling 37. In addtion, they also found that the joint repair effects might be related to exosomal CD73 expression which can convert extracellular AMP to adenosine, as well as exosomal transforming growth factor-β (TGF-β) and insulin growth factor (IGF).

Due to the feasibility to obtain human infrapatellar fat pad from OA patients by arthroscopy, using exosomes from adipose-derived mesenchymal stem cells (ADSCs) has gradually gain more attentions. Tofiño-Vian *et al* found exosomes from ADSCs could down-regulated senescence-associated β-galactosidase activity and the accumulation of γH2AX foci, and reduced the production of inflammatory mediators from OA osteoblasts 38. They also reported that exosomes from ADSCs could reduce the production of inflammatory and catabolic mediators from OA chondrocytes stimulated with IL-1β 39. The chondroprotection role could be the consequence of a lower activation of nuclear factor-κB and activator protein-1. In other study, Wu *et al* attributed the cartilage protection of exosomes from ASCs to the high level of exosomal miR-100-5p 40, because the exosomal miR-100-5p could bind to the 3′-untranslated region (3'UTR) of mTOR, then significantly enhance autophagy level in OA chondrocytes via mTOR inhibition.

#### Therapeutic contents in exosomes

It is significant to figure out the therapeutic contents in exosomes. Recently, Liu *et al* verified that lncRNA KLF3-AS1 in human MSCs and exosomes derived from human MSCs (MSC-Exos) by qRT-PCR analysis 41, and it might be the key molecule with therapeutic effect. In their study, treating rat chondrocytes with the MSC-Exos whose lncRNA KLF3-AS1 expression was knocked down could reverse the normal chondroprotection of MSC-Exos. The knee joint cartilage damage of rat OA model was also deteriorated by the MSC-Exos without lncRNA KLF3-AS1 expression. These data suggested that the therapeutic effect of MSC-Exos on OA is related to the newly discovered exosomal lncRNA KLF3-AS1.

#### Comparison of the effects of exosomes

More importantly, exosomes from different cell types may have different effects, and this topic is still under investigation. Zhu *et al* compared the effects of exosomes secreted by synovial membrane MSCs (SMMSC-Exos) and exosomes secreted by induced pluripotent stem cell-derived MSCs (iMSC-Exos) in treating OA 42. They found both exosomes attenuated OA in the mouse OA model, but iMSC-Exos had a superior therapeutic effect compares to SMMMSC derived exosomes, and iMSC-Exos exerted a stronger effect on chondrocyte migration and proliferation *in vitro*. In another study, Chen *et al* found that exosomes derived from chondrocytes (CC-Exos) increased collagen deposition and minimized vascular ingrowth in engineered constructs, and efficiently and reproducibly developed into cartilage, while the BMSC-Exos treated tissue engineered construct was characterized with hypertrophic differentiation accompanied by vascular ingrowth 43. *In vitro*, CC-Exos could stimulate cartilage progenitor cells proliferation and significantly promoted chondrogenesis-related factors at the mRNA and protein levels.

### Exosomes from molecular engineered cells

**Figure 3:**
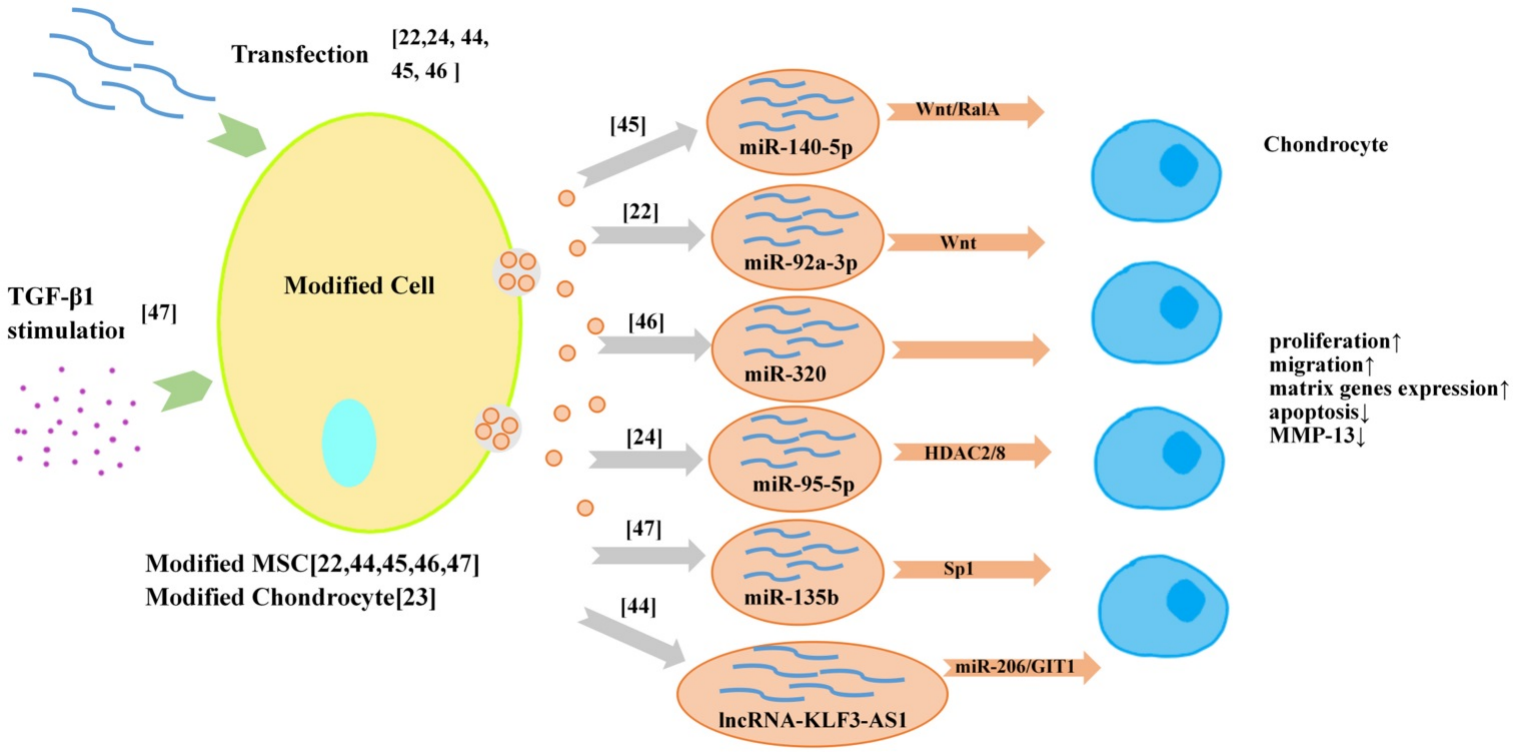
Exosomes from modified cells for OA therapy/cartilage repair.

The strategies of utilizing exosome loading technology to obtain customized drug-loaded exosomes are gradually applied in the field of OA therapy (**Figure 3**). There are two ways to load drugs into exosomes, one is to load drugs into the donor cell of exosomes, such as using transfection and co-incubation; the other is to load drugs into exosomes after they are secreted, such as direct mixing, which the loading efficacy is a big concern. At present, most researchers prefer to obtain the exosomes for OA therapy with high expression of miRNA or lncRNA from modified MSCs 22,44-46. Exosomal miR-92a-3p 22, exosomal lncRNA-KLF3-AS1 44, exosomal miR-140-5p 45 and exosomal miR-320c 46 from transfected MSCs have been reported to have significant therapeutic effects on OA *in vivo* and* in vitro*. Furthermore, it was reported that primary chondrocytes also could be modified to serve as the donor cells of exosomes 24. In that study, exosomes derived from miR‐95‐5p‐overexpressing primary chondrocytes promoted cartilage developpment and cartilage matrix expression by directly targeting HDAC2/8 in MSCs induced to undergo chondrogenesis and chondrocytes, respectively. In addition to transfection, treating the donor cells with proper growth factors such as TGF β1 can also improve the productivity of therapeutic exosomes 47.

## Tissue engineering strategies for exosomes

**Figure 4:**
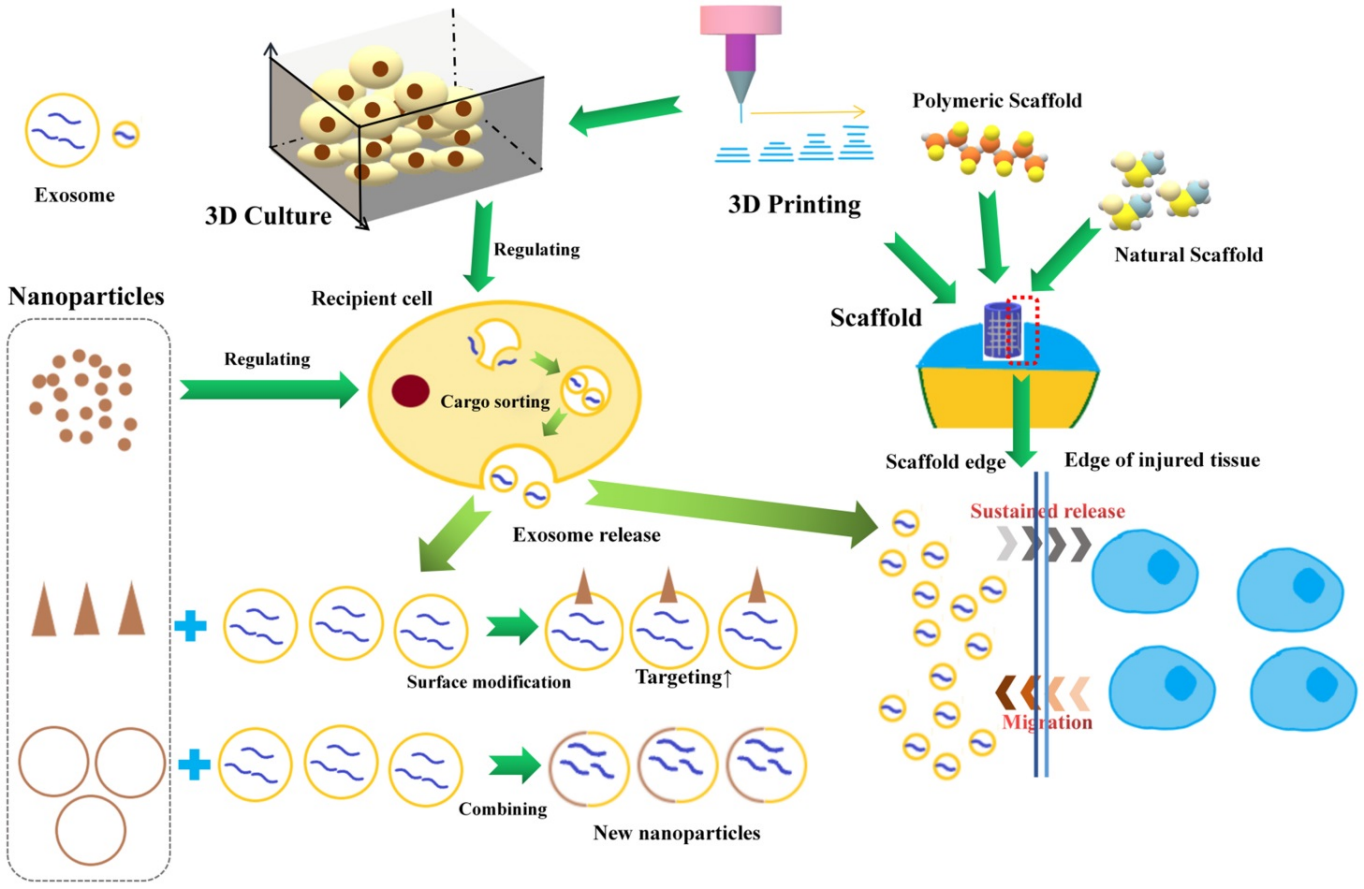
Exosome-based engineering strategy.

Exosome-based tissue engineering technology representing an advanced strategy, has attracted increasing attention in many fields (**Table 1**). In this section, we will focus on some representative exosome-based tissue engineering strategies (**Figure 4**) and discuss their application potential in OA therapy/cartilage repair.

### 3D culture for exosome generation

In a multicellular organism, tissue cells are highly organized in a 3-dimensional (3D) fashion and are surrounded by the extracellular matrix (ECM) 48. Under 2-dimensional (2D) conditions, cells lack the *in vivo* spatial polarization and architecture, leading to changes in cellular morphology, proliferation and functionalities, such as the processing and function of EVs 49. Therefore, for better understanding the roles of exosomes in OA development and treatment, the technology of 3D culture, has attracted increasing attention in order to improve on the inadequate reproduction of the *in vivo* microenvironment by 2D culture.

Some studies have reported that exosome produced by 3D cultures (Exos/EVs-3D) has more advantages than exosome produced by 2D cultures (Exos/EVs-2D) 50-57 in terms of the size, content, function and production efficiency of exosomes. The functions of exosomes differ according to their content, while Exos/EVs-3D contain a different profile of proteins and genetic materials compared to Exos/EVs-2D 52. The enhanced production efficiency of Exos/EVs-3D might relate to the reduction of expression of F-actin in 3D culture 53. However, there is also one study reporting no difference in functions regarding the 2D/3D derivation of exosomes 54.

### Nanoparticles

Nanoparticles can influence and assist the production and function of exosomes. Kasper *et al* found silica nanoparticles could decrease secretion of ICAM/E-selectin bearing exosomes/microvesicles when exposed to the inflamed endothelium 58. Roma-Rodrigues *et al* found that gold nanoparticles (AuNPs) functionalized with thiolated oligonucleotides anti-RAB27A could decrease the release of exosomes due to specific gene silencing 59. In addition, nanoparticles can also affect the sorting of exosomal cargos. Liang *et al* demonstrated that Sphk2 gene silencing induced by siRNA loaded nanoparticles could reduce miRNA-21 sorting into exosomes 60. Nanoparticles can also enhance the targeting ability of exosomes. Khongkow *et al* reported that the surface modification of AuNPs with brain-targeted exosomes derived from genetically engineered mammalian cells enhanced their transport across the blood-brain-barrier 61. Moreover, nanoparticles can also enhance the drug loading ability of exosomes. In order to solve the problem of low efficiency of exosomes in encapsulation of large nucleic acids, Lin *et al* developed a kind of hybrid nanoparticle combining exosome and liposome *via* simple incubation, which efficiently encapsulates large plasmids 62.

### 3D biomaterials and exosome retention

3D scaffolds could be further used as working platforms for the exosomes while attempting to control the release of exosomes in the tissue repaired area 63-64. Hydrogel has been widely used as 3D scaffold due to their unique features, such as high water content, biocompatibility, swelling behavior, and modulated 3D networks, in many restorative areas, such as cardiac repair 65, vascular disease 66, wound healing 67. Recently, Zhang *et al* reported that chitosan hydrogel could notably increase the stability of proteins and miRNAs in exosomes 68. In OA therapy, hydrogel materials also have been widely to better fill the cartilage defect and provide a mode and mechanical support for cartilage regeneration 69. Interestingly, hydrogel materials have been proven to have good exosome retention and sustained release function. Schneider *et al* found that many types of proteins secreted by chondrocytes encapsulated within photoclickable poly(ethylene glycol) hydrogels have been reported to be present within cell-secreted exosomes 70. They suggested that the ability of diffusing through the hydrogel of smaller exosomes contributed to the results. Liu *et al* exploited a photoinduced imine crosslinking hydrogel glue as an exosome scaffold to prepare an acellular tissue patch for cartilage regeneration 71. They found that most of the encapsulated exosomes were retained inside the hydrogel (>90%) after immersing in PBS for 14 days. In addition, the tissue patch could release low concentration of exosomes showing positive regulation to the surrounding cells.

### 3D bio-printing

3D printing has been well used in cartilage tissue repair, and to adopt the great bioeffects of exosome into 3D printing technology, there are two break points for further development. One is to improve the productivity and functions of exosomes by using optimized 3D culture microenvironment, which can be precisely designed and printed with 3D bio-printers 57. The other is to design advanced bio-mimic scaffolds with more optimized geometric structure and better incorporate with exosomes, thus enhancing the therapeutic effects of exosomes or EVs 72-73. For instance, Chen *et al* reported the interaction between 3D printing scaffold and exosome in cartilage repair 74. They fabricated a 3D printed cartilage ECM/gelatin methacrylate/exosome scaffold with radially oriented channels using desktop-stereolithography technology. They found the 3D printed scaffold could effectively retain exosomes for 14 days *in vitro* and could retain exosomes for at least 7 days *in vivo*. They also found that the 3D printed scaffold could recruit chondrocytes, which was mainly attributed to ECM, and that exosomes could further enhance this effect.

## Future perspective

### Drug-loading techniques

The cell-friendly biological feature of exosome decides that the future drug application *via* exosome may provide a higher delivery efficiency. The recent research tend is to modify the exosome-secreting cells with transfection to load miRNA(s) into exosomes, thus can be used for OA therapy/cartilage repair 23,44-46. However, the transfection method in loading RNA has many limitations, such as the unstable productivity of RNA and the unclear factors influencing the RNA level and loading 75, which means the application is still far from clinic. Other drug loading techniques applied for OA treatment/cartilage repair have not been reported yet, therefore it is significant to develop new drug loading technologies. One of the direction is to use nanoparticles, which could improve the drug loading capacity of exosomes 62. All in all, to explore the cargo-loading mechanism of exosomes and search for more efficient and stable drug loading technology, will accelerate the development of exosome-based OA therapy/cartilage repair.

### Local sustained release system

Most recent studies demonstrate that exosomes play a significant role in OA therapy/cartilage repair both in animal models and preclinical trials. Nevertheless, the current therapy requests repeatedly local administration for maintaining the effective concentration, which increase pain and the risk of side effects to the subjects. To solve this problem, local controlled release of exosomes with bio-scaffold has begun to be valued 74. Controlled delivery of therapeutic agents on local joint lesion possesses two advantages. One is that controlling the degradation time of drug vehicle/bio-degradable scaffold and maintaining the effective concentration of drugs, thus prolonging the functional time of drugs at desired dosages. The other is that it will be helpful to combine the exosome-working-platform with other sustained drug release technologies according to the degrees of cartilage injury and osteoarthritis, which can be designed and become personalized therapeutics in the future.

### Personalized and “point-to-point” treatment

Over the past few years, the arrival of liquid biopsy technology has made the generating a database of OA patients much easier, including information concerning exosomes. With the gradual understanding of the mechanism of exosome involved in OA and adequate information from the database, the patient's articular condition can be analyzed individually. It would be possible to assemble the needed cargos and drugs into the modularized exosomes to achieve personalized treatment with maximizing the therapeutic effect. In addition, the key concept of precision medicine is to treat or “ablate” the pathological condition without damage to normal tissues. As a consequence, exosomes will be a poetical biomarker and therapeutic tool for personalized and precision treatment of OA therapy/cartilage repair in the future.

## Conclusion

Exosomes derived from chondrocytes, synovial cells, and synovial fluid have been shown to be involved in the pathogenesis of OA. Meanwhile many studies have shown that exosomes from natural cells, especially MSCs, could maintain chondrocyte homeostasis and ameliorate the pathological severity of OA, demonstrating the potential therapeutic effect of exosomes for OA/cartilage injury. In addition, exosomes from modified cells with drug loading technologies have been shown improved therapeutic effect. Tissue engineering techniques are also used in the exosome-based OA therapy/cartilage repair. Biological scaffolds, especially hydrogel, have been shown to have a good sustained exosome-release effect in cartilage repair. The 3D printing technology can be used to construct more reasonable 3D culture microenvironments for exosomes, and can contribute to design scaffolds with more optimized geometric structure. We believe that drug loading, sustained release and individualized treatment will be the main directions of exosome-based OA therapy/cartilage repair in the near future.

## Figures and Tables

**Table 1:** Tissue Engineering Strategies for Osteoarthritis and Cartilage Injury

Tissue-Engineering Technology	Advantages for exosomes	Related reports
3D culture	Regulating the biological characteristics;	Size^50^-^51^; Content^50^-^52^, ^55^-^57^; Function^50^, ^52^, ^54^-^55^; Production efficiency^50^, ^52^-^53^, ^55^-^57^
Nanoparticles	Changing biological characteristics	Regulating exosome release of parent cells 58,59; Regulating content of exosome^60^;
	Surface modification;	Improving the targeting ability of exosomes^61^;
	Combining with exosomes for improving deficiencies;	Efficiently encapsulating large plasmids^62^;
3D biomaterials	Exosome retention and sustained release function as working platforms;	Bone regeneration^63^; Cardiac repair^65^; Vascular disease^66^; Wound healing^67^; Cartilage repair^71^;
	Increasing the stability of content;	Increasing the stability of proteins and miRNAs in exosomes^68^;
3D printing	Designing more optimized 3D culture microenvironment;	Improving the productivity and functions of exosomes^57^;
	Designing scaffolds with more optimized geometric structure;	Bone defect repair^72^; Osteoangiogenesis^73^; Cartilage repair^74^;
